# Surgical decision-making in paediatric penetrating trauma: case report from two paediatric tertiary centres

**DOI:** 10.3389/fsurg.2026.1810768

**Published:** 2026-04-17

**Authors:** Simone Frediani, Lorenzo Nanni, Martina Granello, Ilaria Buconi, Angelo Zarfati, Filomena Valentina Paradiso, Antonella Accinni, Sara Silvaroli, Arianna Bertocchini, Federico Beati, Silvia Madafferi, Cristina Martucci, Valerio Pardi, Ivan Pietro Aloi

**Affiliations:** 1 Pediatric General and Urgency Surgery Unit, Bambino Gesù Children's Hospital IRCCS, Rome, Italy; 2 Pediatric Surgery, IRCCS, Policlinico Universitario A. Gemelli, Rome, Italy

**Keywords:** computed tomography angiography, contrast-enhanced computed tomography, haemodynamically stablepatients, pediatric trauma, penetrating wounds, selective surgical exploration, surgical decision making, surgical decision-making

## Abstract

**Introduction:**

Penetrating trauma in children is relatively uncommon but is associated with significant morbidity and mortality, particularly when major vascular or visceral structures are involved. Owing to anatomical and physiological differences, as well as limited paediatric-specific evidence, surgical decision-making remains challenging and often relies on extrapolation from adult data. This study aimed to describe the surgical decision-making strategies for haemodynamically stable paediatric patients with penetrating injuries, highlighting the roles of clinical assessment, imaging, and multidisciplinary management.

**Methods:**

We report a retrospective case series of three paediatric patients with penetrating trauma who were managed at two tertiary paediatric referral centres. The clinical presentation, diagnostic workup, surgical approach, and outcomes were analysed.

**Case description:**

All patients were haemodynamically stable on admission but presented with penetrating injuries involving high-risk anatomical regions. Contrast-enhanced computed tomography played a key role in the preoperative assessment of extremity injuries, whereas surgical exploration was deemed mandatory in the presence of abdominal evisceration, despite stable vital signs. A tailored surgical approach based on clinical and radiological findings allowed safe foreign body removal or exploratory surgery without major complications. No vascular or visceral injuries requiring repair were observed. The postoperative course was uneventful, and no early or late complications occurred during follow-up.

**Conclusion:**

Penetrating trauma in haemodynamically stable paediatric patients requires individualised decision-making, supported by careful clinical evaluation, appropriate imaging, and multidisciplinary collaboration. Selective surgical exploration guided by injury pattern and anatomical risk can result in favourable outcomes while avoiding unnecessary procedures.

## 1 Introduction

Trauma remains the leading cause of mortality and long-term disability in the paediatric population (1). Although penetrating trauma accounts for a smaller proportion of injuries in children compared with adults, it is associated with disproportionately high morbidity and mortality, particularly when major vascular or visceral structures are involved. Gunshot wounds, stab injuries, and impalements are the most common mechanisms of penetrating trauma in paediatric patients, with significant variability in injury patterns and outcomes (2–4).

Paediatric penetrating trauma poses unique challenges owing to anatomical and physiological differences, limited circulating blood volume, and the rarity of these injuries, which restricts the availability of high-quality paediatric-specific evidence. As a result, management strategies are often extrapolated from adult trauma protocols despite important differences in injury mechanisms, tissue elasticity, and healing potential.

Decision-making in penetrating trauma is primarily guided by haemodynamic status, anatomical location of the injury, and clinical signs of vascular or visceral involvement. Although immediate surgical exploration is mandatory in unstable patients or those with hard signs of vascular injury, the optimal management of haemodynamically stable children remains controversial. Advances in imaging, particularly contrast-enhanced computed tomography (CT), have enabled more selective and tailored approaches, potentially reducing unnecessary surgical interventions.

However, variability in institutional expertise, the availability of multidisciplinary teams, and injury characteristics continue to influence clinical practice. Sharing detailed clinical experiences may help refine decision-making strategies and improve outcomes in this vulnerable population. This study aimed to describe and analyse surgical decision-making in haemodynamically stable paediatric patients with penetrating trauma, focusing on the role of clinical assessment, imaging, and multidisciplinary management in guiding selective surgical interventions.

## 2 Cases description

### 2.1 Case 1

**Figure 1:**
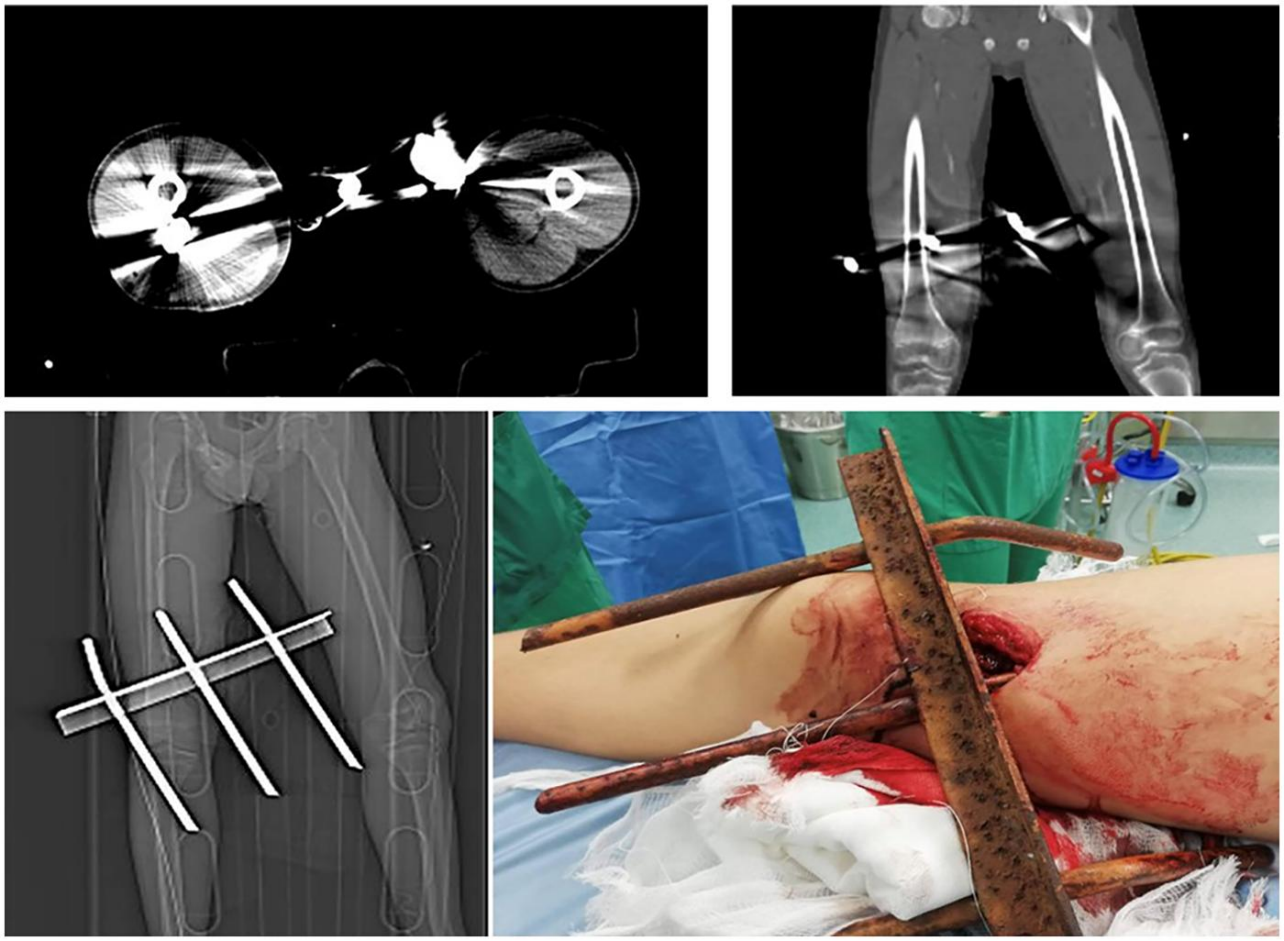
Clinical and radiological findings of Case 1.

A 12-year-old boy with no relevant medical history was transported to our Emergency Department by helicopter following a penetrating injury to the right thigh. The injury occurred when the patient attempted to climb over an iron gate, slipped, and sustained penetration of the anteromedial thigh by a metallic bar that remained *in situ* (Figure 1). The prehospital personnel immobilised the affected limb, applied a protective dressing, and avoided manipulating the foreign body to minimise the risk of haemorrhage and tissue damage. Upon arrival, the patient was alert and haemodynamically stable. Physical examination revealed a metallic rod penetrating the mid-portion of the right thigh, with no active arterial bleeding, expanding haematoma, or clinical signs of compartment syndrome. The distal pulses were palpable and symmetrical, and the motor and sensory functions of the right lower limb were preserved. No additional injury was observed. The patient was admitted to the critical care unit for trauma management. Tetanus prophylaxis was administered using human tetanus immunoglobulin and a booster dose of the tetanus vaccine, according to institutional protocol. Initial laboratory tests showed a haemoglobin level of 13.7 g/dL and a normal white blood cell count, with no biochemical evidence of acute blood loss or infection. Contrast-enhanced CT of the right lower limb was performed within 30 min of admission and demonstrated a metallic foreign body traversing the thigh from the anteromedial to posterolateral compartments. The rod was located close to the distal third of the femoral diaphysis, without cortical disruption. The arterial and venous structures were patent, with no evidence of vascular injury, contrast extravasation, or pseudoaneurysm formation. Air bubbles were noted within the posterior thigh compartment, extending toward the popliteal fossa, which was consistent with soft tissue trauma.

Given the patient's haemodynamic stability and absence of vascular or skeletal injury, surgical removal of the foreign body and exploration of the wound tract were performed. In the operating room, after removing the external dressing, the metallic foreign body was exposed, with no evidence of active bleeding or associated lesions. Following antiseptic preparation with povidone–iodine, the foreign body was removed under direct visualisation without haemorrhage or changes in distal pulse oximetry. Exploration of the wound tract revealed a non-bleeding laceration of the quadriceps muscle belly. The wound was extensively irrigated with a diluted povidone–iodine solution, and an 8-Fr corrugated drain was placed along the tract to prevent fluid accumulation and reduce the risk of deep infection. Layered closure was performed, and the skin was sutured with interrupted 3–0 Prolene sutures. The postoperative course was uneventful. The patient received intravenous ceftriaxone for 5 days, followed by oral cefixime for an additional 7 days. Haemoglobin levels and white blood cell counts remained stable throughout the hospitalisation. The surgical site remained clean, and no signs of fever, haematoma, or local or systemic infection were observed. The patient was discharged in good clinical condition, with a corrugated drain *in situ* and instructions for outpatient follow-up.

On postoperative day 10, outpatient evaluation revealed a well-healed surgical wound without signs of infection, and the corrugated drain was safely removed. At the most recent follow-up, the patient remained asymptomatic, with no late complications.

### 2.2 Case 2

A 16-year-old boy with no relevant medical history was brought to our Emergency Department by ambulance after sustaining a stab wound to the left upper abdomen during an assault. No pre-hospital interventions beyond basic wound coverage were required. On arrival, the patient was alert, cooperative, and in good general condition. The patient's vital signs were stable, with a heart rate of 58 beats per min, blood pressure of 99/68 mmHg, oxygen saturation of 96% on room air, and normal peripheral pulses. The patient was eupnoeic and presented with localised abdominal pain, without associated nausea or vomiting. Physical examination revealed a soft, non-distended abdomen, without guarding or rebound tenderness, and no clinical signs of peritonitis. A penetrating wound, approximately 3 cm in length, was visible in the left hypochondrium, with protrusion of omental fat and small bowel loops through the abdominal wall defect. The surrounding skin showed no signs of active bleeding or gross contamination. No additional traumatic injuries were identified during the secondary survey. Initial laboratory investigations revealed a haemoglobin level of 15 g/dL and a white blood cell count of 8.6 × 10⁹/L, with normal coagulation and serum electrolyte levels. Abdominal ultrasonography revealed no abnormalities of the liver, pancreas, spleen or kidneys. Free intraperitoneal fluid was not detected in the accessible abdominal recesses. Despite haemodynamic stability and the absence of ultrasonographic signs of intra-abdominal injury, the presence of bowel and omental eviscerations raised significant concerns regarding peritoneal violation. Surgical exploration was performed to exclude occult visceral injuries. The patient was taken to the operating room for an exploratory laparotomy. Intraoperatively, an oblique epigastric wound, approximately 6 cm in length, was identified. A full-thickness laceration of the left rectus abdominis muscle was noted cranially, extending caudally to the right rectus muscle. A 3-cm breach of the peritoneum exposed the peritoneal cavity. The incision was extended to allow adequate exposure, and a systematic exploration of the abdominal cavity was performed. Inspection of the hepatic surface, gallbladder, medial border of the spleen, stomach, and the first and second portions of the duodenum revealed no traumatic lesions. The ascending, transverse and descending colon remained intact. The entire small bowel was carefully examined from the duodenojejunal junction to the ileocaecal valve. No perforations, serosal tears, mesenteric injuries, or intramural haematomas were observed. No active bleeding or peritoneal cavity contamination was observed. The abdomen was thoroughly irrigated with warm saline, haemostasis was confirmed, and no drain was deemed necessary. The abdominal wall was closed in layers, and the skin was sutured using interrupted Prolene sutures. The postoperative course was uneventful. Adequate pain control was achieved with standard analgesic therapy. Oral intake was resumed on postoperative day 1 with good tolerance, and normal bowel function progressively returned. The patient received intravenous ceftazidime for 3 days, followed by oral cefixime for an additional 7 days. The haemoglobin levels measured on postoperative days 1 and 3 remained stable. No postoperative complications occurred, and the patient was discharged in good clinical condition on postoperative day 3. At the 10-day outpatient follow-up, the surgical wound appeared clean, with no signs of infection or dehiscence, and the skin sutures were removed without complication. Follow-up has remained uneventful to date.

### 2.3 Case 3

**Figure 2:**
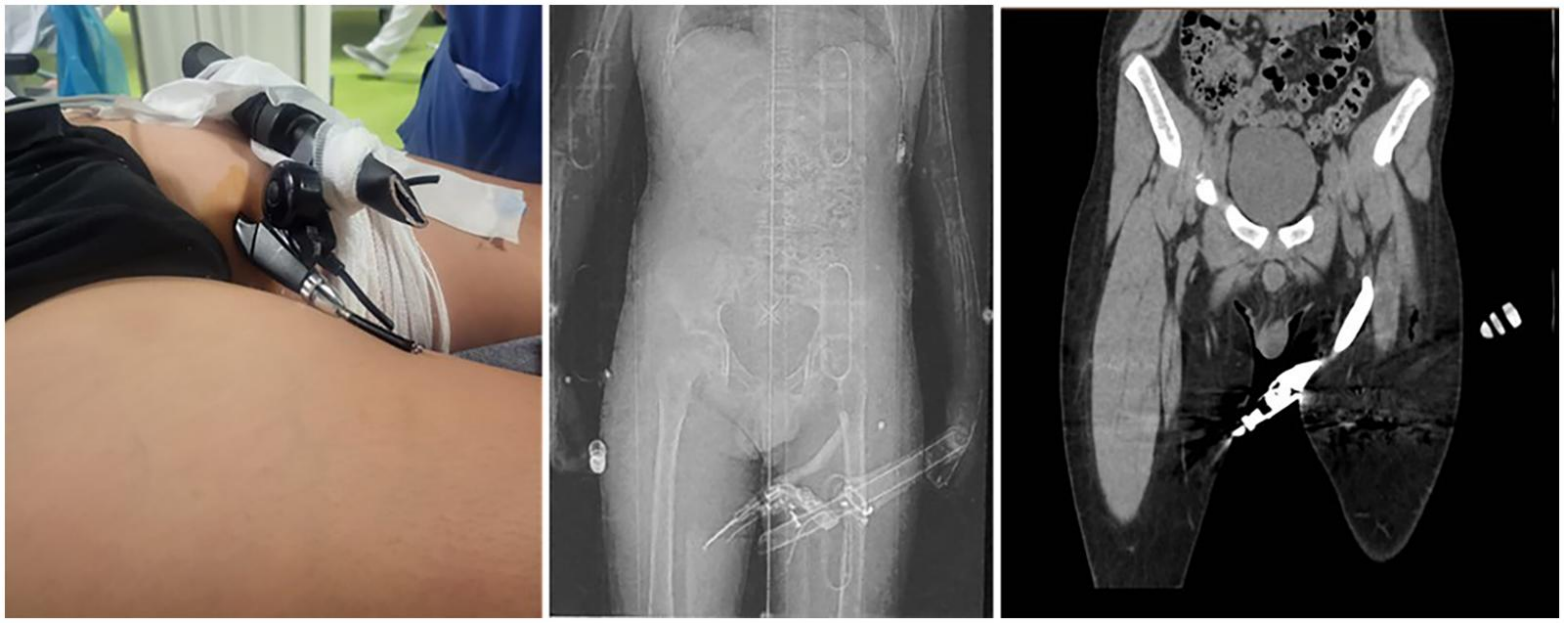
Clinical and radiological findings of Case 3.

A 12-year-old boy was admitted to the emergency department following an accidental fall from an electric scooter, resulting in a penetrating injury to the anteromedial aspect of the proximal third of the left thigh caused by the brake lever (Figure 2). First, the responders cut the handlebars to extricate the patient and immobilised him using a cervical collar and spinal board. No loss of consciousness was observed. Prehospital management included administration of analgesics, anti-haemorrhagic agents, and antiemetics. Upon arrival, the patient was alert, oriented, and haemodynamically stable, with normal vital signs and no neurological deficits. Active bleeding was not observed. Owing to the mechanism of injury, wound location, and proximity to major vascular structures, a total-body CT was performed to assess the extent of trauma. Imaging revealed that the distal tip of the metallic foreign body was adjacent to the superficial femoral artery, causing indentation of the lateral wall. No haematoma or active contrast extravasation was identified; however, perivascular fat stranding suggested minimal haemorrhagic infiltration. Based on clinical and radiological findings, emergency surgery was performed in collaboration with vascular and orthopaedic surgeons. After antiseptic preparation, an oblique incision was made at the exit site of the foreign body, located at the medial root of the thigh. The brake lever was carefully removed without causing bleeding. The wound was irrigated with povidone-iodine, and a Penrose drain was placed within the fascial defect. The layered closure was completed. The patient was admitted to the paediatric intensive care unit for 24 h and then transferred to the paediatric surgery ward. Postoperative recovery was smooth, with preserved motor and sensory functions and adequate pain control. Full weight-bearing mobilisation was permitted. The patient was discharged on postoperative day 3 with oral amoxicillin-clavulanate (1 g, twice daily for 7 days) and analgesics. After 7 days, the Penrose drain was removed, and an Argentum Aquacel dressing was applied. The sutures were removed 10 days after the injury, with complete wound healing. Follow-up has remained uneventful to date.

The main clinical characteristics, diagnostic findings, and surgical management of the cases are summarised in Table 1. A detailed clinical timeline of the three cases is summarised in Table 2.

**Table 1:** Clinical characteristics, diagnostic findings and surgical management of the reported cases.

Case	Age (years)	Mechanism	Site	Imaging	Surgery	Hospital Stay (Days)	Outcome
1	12	Impalement on iron gate	Right thigh	CT	Surgical removal and wound exploration	10	Complete recovery
2	16	Stab wound	Left upper abdomen	US	Exploratory laparotomy	3	Complete recovery
3	12	Scooter Brake Lever Impalement	Left Thigh	CT	Surgical Removal And Wound Exploration	3	Complete Recovery

**Table 2:** clinical timeline and management of the reported cases.

Phase	Case 1	Case 2	Case 3
Prehospital Management	Limb immobilisation, foreign body left *in situ*	Basic wound coverage	Handlebar cut to free patient, immobilisation
Emergency room admission	Haemodynamically stable, intact distal pulses	Haemodynamically stable, bowel and omental evisceration	Haemodynamically stable, no neurological deficit
Imaging	Contrast-enhanced CT of the thigh	Abdominal ultrasound	Whole-body CT
Key Findings	No vascular injury	Suspected peritoneal violation	Foreign body close to superficial femoral artery
Surgical Treatment	Foreign body removal and wound exploration	Exploratory laparotomy	Foreign body removal with vascular team standby
Postoperative Course	Uneventful	Uneventful	Uneventful
Discharge	With drain *in situ* on day 7. Drain removed day 10.	Postoperative day 3	Postoperative day 3
Follow-Up	Complete recovery	Complete recovery	Complete recovery

## 3 Discussion

Penetrating trauma is a relatively uncommon mechanism of injury in the paediatric population, but it is associated with substantial morbidity and mortality, particularly when major vascular or visceral structures are involved. Paediatric patients account for approximately 20% of emergency department visits (4); however, penetrating injuries constitute only a small proportion of paediatric trauma admissions. Despite their relative rarity, these injuries pose significant diagnostic and therapeutic challenges owing to anatomical and physiological differences between children and adults, limited circulating blood volume, and the reduced distance between the skin surface and vital organs in younger patients. Consequently, the mortality risk is higher in younger children than in adolescents, largely because of their smaller body frames and the close proximity of critical structures (1).

The management of penetrating trauma in paediatric patients remains complex and heterogeneous. Considerable variability exists among trauma centres regarding institutional expertise, clinical experience, and trauma team composition (2). Moreover, high-quality paediatric-specific evidence is limited, and many current management strategies continue to be extrapolated from adult trauma protocols. This lack of standardised paediatric guidelines significantly complicates clinical decision-making, particularly for haemodynamically stable patients, in whom the indication for operative vs. non-operative management remains controversial (5).

Haemodynamic status remains the cornerstone of initial assessment and management. Immediate operative exploration is universally indicated in children presenting with persistent haemodynamic instability or haemorrhagic shock unresponsive to resuscitation, as well as in life-threatening conditions requiring immediate control, such as cardiac tamponade or massive haemorrhage (1). Similarly, impalement injuries should not be treated outside the operating room, as premature removal may precipitate catastrophic bleeding, and trimming of the object may be necessary only to facilitate safe transport and positioning (1, 6).

Conversely, most paediatric patients with penetrating trauma are haemodynamically stable, and decision-making in this subgroup is considerably more nuanced. Children exhibit remarkable physiological compensatory mechanisms that may delay hypotension despite significant blood loss, thereby masking early haemorrhagic shock (1, 7). Therefore, management decisions must be individualised and supported by frequent clinical reassessments and multidisciplinary discussions.

Penetrating abdominal trauma is one of the most debated areas in paediatric trauma care. Historically, diagnostic peritoneal lavage and mandatory laparotomy were widely performed. With advances in minimally invasive surgery and imaging, more selective strategies have emerged. Diagnostic laparoscopy may be useful in detecting peritoneal violations and avoiding unnecessary laparotomies in selected cases (1). However, once peritoneal penetration is confirmed, particularly in the presence of bowel or omental evisceration, operative exploration remains indicated, regardless of haemodynamic stability.

CT is currently the most accurate imaging modality for evaluating penetrating abdominal trauma. Dual-phase contrast-enhanced CT allows detailed assessment of the wound trajectory, solid organ injury, and associated vascular or skeletal damage (8, 9). However, its sensitivity to hollow viscus and mesenteric injuries remains limited. Inaba et al. reported a sensitivity of only 31.3% for hollow viscus injury detection on CT, highlighting that a negative scan cannot reliably exclude clinically significant bowel injury (4, 10). Consequently, imaging findings must always be interpreted in conjunction with the clinical evaluation. In the present series, operative exploration was justified despite stable physiological parameters because of the high-risk nature of abdominal evisceration.

The management of extreme penetrating trauma has evolved substantially with the widespread adoption of advanced imaging techniques. Computed tomography angiography (CTA) is considered the first-line diagnostic modality for haemodynamically stable patients with suspected vascular injuries (1, 11). CTA enables the accurate evaluation of arterial patency, detection of foreign bodies, assessment of fracture patterns, and identification of soft-tissue emphysema, facilitating safe and targeted surgical planning (9).

Children presenting with a normal physical examination and ankle–brachial indices ≥0.9 may be safely observed, whereas symptomatic patients or those with indices <0.9 generally require angiographic evaluation (1). Importantly, in the presence of hard signs of vascular injury—such as active bleeding, expanding haematoma, bruit or thrill, or distal ischaemia—current guidelines recommend immediate vascular surgery consultation and prompt operative intervention without delay for imaging studies (2).

Optimal management of paediatric penetrating trauma requires a coordinated multidisciplinary approach involving paediatric, vascular, and orthopaedic surgeons, anaesthesiologists, and interventional radiologists. Outcomes are strongly influenced by paediatric-specific expertise, including familiarity with appropriately sized instruments, weight-based medication dosing, and specialised perioperative care (1, 11). Postoperative management should include close neurovascular monitoring, preferably in an intensive care setting, particularly after vascular injury or reconstruction. Posttraumatic anticoagulation strategies remain insufficiently studied in children and should therefore be individualised (11).

This study had some limitations, including the retrospective design, small sample size, heterogeneity of injury mechanisms, and limited follow-up duration. Nevertheless, detailed case-based analyses remain valuable in rare clinical scenarios in which randomised or prospective studies are difficult to conduct.

In the absence of robust evidence-based criteria, selective management strategies have progressively replaced mandatory surgical exploration in paediatric patients with penetrating trauma (9, 10). However, decision-making continues to rely on the careful integration of clinical findings, imaging results, anatomical injury patterns, and institutional expertise. The cases presented in this series support a selective, risk-based, and patient-centred approach, emphasising the importance of individualised management and multidisciplinary collaboration. Further multicentre studies are required to improve risk stratification and to develop standardised paediatric-specific guidelines capable of optimising outcomes while minimising unnecessary surgical interventions (5).

In conclusion, although uncommon, penetrating trauma in children requires a careful and individualised approach. In haemodynamically stable patients, clinical evaluation combined with appropriate imaging allows for tailored decision-making and selective surgical exploration. Extremity injuries may be safely managed with imaging-guided surgical planning, whereas abdominal evisceration remains an indication for operative exploration, regardless of haemodynamic stability. Multidisciplinary collaboration is essential for optimising outcomes. This case series supports a selective, patient-centred approach to paediatric penetrating trauma, to minimise morbidity while ensuring patient safety.

## Data Availability

The original contributions presented in the study are included in the article/Supplementary Material, further inquiries can be directed to the corresponding author.
